# Macrophage Polarization: Learning to Manage It 3.0

**DOI:** 10.3390/ijms26010311

**Published:** 2025-01-01

**Authors:** Nadia Lampiasi

**Affiliations:** Consiglio Nazionale delle Ricerche, Istituto per la Ricerca e l’Innovazione Biomedica, Via Ugo La Malfa 153, 90146 Palermo, Italy; nadia.lampiasi@irib.cnr.it; Tel.: +39-0916809513; Fax: +39-0916895548

Macrophages are cells of the innate immune system with very peculiar characteristics, so plastic that they respond rapidly to environmental changes by assuming different and sometimes contrasting functions, such as initiating a physiological inflammatory response or interrupting it and repairing damaged tissues. The definition of pro- and anti-inflammatory macrophages (classical M1 and alternative M2) is now outdated. Instead, we observe a continuum of different cell types between the two extremes (M1 and M2) that show some overlapping characteristics (e.g., production of the same cytokines) and others that are completely unique (e.g., production of pro- or anti-inflammatory cytokines and expression of receptors and CD markers). Therefore, macrophages may participate in the same event but with different functions [1,2]. This variety of cell types also includes tumor-associated macrophages (TAMs). TAMs can derive from either resident macrophages or from blood monocyte-derived macrophages (BMDMs) that migrate because they are attracted by factors produced by the tumor itself [3,4]. TAMs are known to promote tumorigenesis and cancer progression, in addition to inducing metastasis formation and resistance to chemotherapy and radiotherapy [5,6]. They constitute approximately 50% of the tumor mass and influence tumor cells (TCs), the tumor microenvironment (TEM), and cancer-associated fibroblasts (CAFs) [7]. TAMs showing the characteristics of the M2d macrophage subtype have been identified in the ascites of patients with ovarian cancer [7]. The repolarization or reprogramming strategy of TAMs is based on the well-known plasticity of macrophages and, therefore, on the possibility of redirecting the M2-like phenotype towards the M1-like phenotype, which can produce pro-inflammatory cytokines and chemokines directed against cancer [8]. To educate TAMs in a pro-inflammatory direction, it is necessary to manipulate the molecular nodes that distinguish the two main macrophage subtypes (M1 and M2). An interesting manuscript in this Special Issue addresses the role of PARylation in the polarization of TAMs [9]. The authors investigate poly(ADP-ribose) polymerase (PARP) enzymes, in particular, PARP14, which plays a role in breast cancer-induced TAM polarization. The results indicate the involvement of lipocalin-2 (LCN2), macrophage migration inhibitory factor (MIF), and plasminogen activator inhibitor-1 (PAI-1) in TAM polarization and suggest PARP14 as a possible target for TAM repolarization [9] (Figure 1).

To better understand the interactions between TAMs, tumor cells, and TME cues, 3D cell culture models have been developed (for a review, see [10]). Spheroids are a very simple 3D cell culture model for the study of cell–environment interactions. An interesting study explored the reprogramming of BMDMs under 3D free-floating conditions in the presence of breast cancer spheroids [11]. Tumor cell spheroids stimulated the production of immunosuppressive M2 markers, as well as pro-tumor cytokines and chemokines. Despite this, the reprogramming of BMDMs produced a mixed macrophage phenotype that expressed both immunosuppressive and immunostimulatory anti-tumor characteristics [11] (Figure 2).

The third study that addresses the repolarization of TAMs concerns a very particular target: V-set immunoglobulin domain-containing 4 (VSIG4) [12], a protein expressed exclusively by macrophages. In the TME, it is specifically expressed by TAMs [13] and is associated with a poor prognosis [14,15,16] (Figure 3).

The authors demonstrate that VSIG4 contributes to promoting the M2-like polarization of TAMs and therefore appears as a target for their reprogramming [12]. Furthermore, VSIG4 blockade also works as a monotherapy, since it can act in combination with the anti-PD-1 target [12]. PD-1 is an immune checkpoint considered a possible molecular target for TAM reprogramming, together with LncRNAs and miRNAs, signaling pathways, receptors, extracellular matrix components, and cytokines/chemokines [17] (Figure 4). The block of immune checkpoints and molecular targets are used to enhance the efficacy of conventional cancer therapies (chemotherapies/radiotherapies). Moreover, the inhibition of strategic pathways for TAM repolarization, such as NF-κB, can also lead to the downregulation of PD-1 checkpoint expression [18].

This Special Issue 3.0 focuses on the involvement of macrophage dysfunction and inflammation in the development of some diseases, their progression, or their worsening. Indeed, there are many pathologies with implications directly or indirectly related to macrophage polarization, ranging from autoimmune diseases to cancer, including all diseases that have chronic inflammation as their substrate [19,20,21,22]. A review in this Special Issue focuses on bioactive phospholipids such as lysophosphatidic acid (LPA) and their physiological and pathological functions, including cell migration, apoptosis, and proliferation [23].

Indeed, LPA is present in human bronchoalveolar lavage fluid (BALF) and increases after allergic inflammation [24]. Allergic asthma is often characterized by chronic airway inflammation, and asthma therapy targets LPA receptor 2 (LPA2). The LPA-producing enzyme autotaxin (ATX) is predominantly expressed in alveolar macrophages and bronchial epithelial cells and converts lysophospholipids (LPLs) to LPA [25]. The ATX/LPA signaling pathway is active in lung fibrosis via LPA receptor 1 (LPA1). It exacerbates chronic inflammation in chronic disease situations by generating cytokines and attracting inflammatory cells into the local tissue environment [26]. In fact, inhibition of the LPA1 receptor is a strategy used in preclinical studies and clinical trials to reduce pathologies that induce pulmonary fibrosis (PF) [27]. The macrophage is the most active cell type in lipid metabolism, and in PF, most of the lipid metabolism genes are altered [28]. In Chronic Obstructive Pulmonary Disease (COPD), there is an increased number of macrophages with phenotypic and functional changes related to their plasticity [29]. The molecular changes underlying PF and pulmonary emphysema (PE) found in COPD patients—including genetic and epigenetic changes—have been elucidated in a recent review [30]. In this Special Issue, a review explored macrophage polarization and function in COPD patients [31].

Functional changes include impaired phagocytosis and high lipid load in macrophages [32]. Inhibition of sphingosine 1-phosphate (S1P) signaling pathways suppresses the phagocytic capacity of alveolar macrophages (AMSs) [33]. A decreased phagocytic capacity is associated with an increased likelihood of pulmonary bacterial colonization by Haemophilus influenzae, Streptococcus pneumoniae, and Moraxella catarrhalis [34,35] and viral colonization in the lungs of COPD patients. As a consequence of the dysfunction of these macrophages, their number increases significantly, but bacterial colonization of the lower airways in COPD patients increases the frequency of exacerbations and the progression of the disease [36,37]. The World Health Organization (WHO) recently published a Tobacco Knowledge Summary (TKS) that highlights that smoking is the main cause of COPD (about 70%) in most developed countries [38]. COPD patients also have a significantly higher risk of developing lung cancer, cardiovascular disease, and type II diabetes [38]. Nicotine is the main psychoactive component of tobacco. Nicotine transiently activates and deactivates monocyte–macrophages, reducing their ability to fight bacterial and viral infections through the induction of interleukin-1 receptor associated kinase-M (IRAK-M) expression, NF-kB-inhibition, and NLRP3-dependent IL-1β production [39]. Macrophages express nicotinic acetylcholine receptors (nAChRs) [40]. The induction of IRAK-M by nicotine is mediated by the 7-nAChR receptor [39]. The 7-nAChR receptor is involved in the activation of the cholinergic anti-inflammatory pathway, helping to mitigate excessive inflammation and maintain host homeostasis [41]. A review in this Special Issue details the cholinergic constituents present in macrophagesin particular, the 7-nAChR signaling pathway—and discusses their role in promoting macrophage polarization toward anti-inflammatory phenotypes [42]. Furthermore, the consequences of viral infections on macrophage inflammatory phenotypes are explored, taking cholinergic mechanisms into account.

Overall, this Special Issue 3.0 provides a collection of reviews and original articles covering different aspects of macrophage polarization, from macrophage reprogramming as a strategy to fight cancer to microenvironment-induced polarization to mitigate inflammation, which is a substrate of some diseases. The various articles thoroughly illustrate the latest progress in understanding some mechanisms underlying macrophage plasticity.

## Figures and Tables

**Figure 1:**
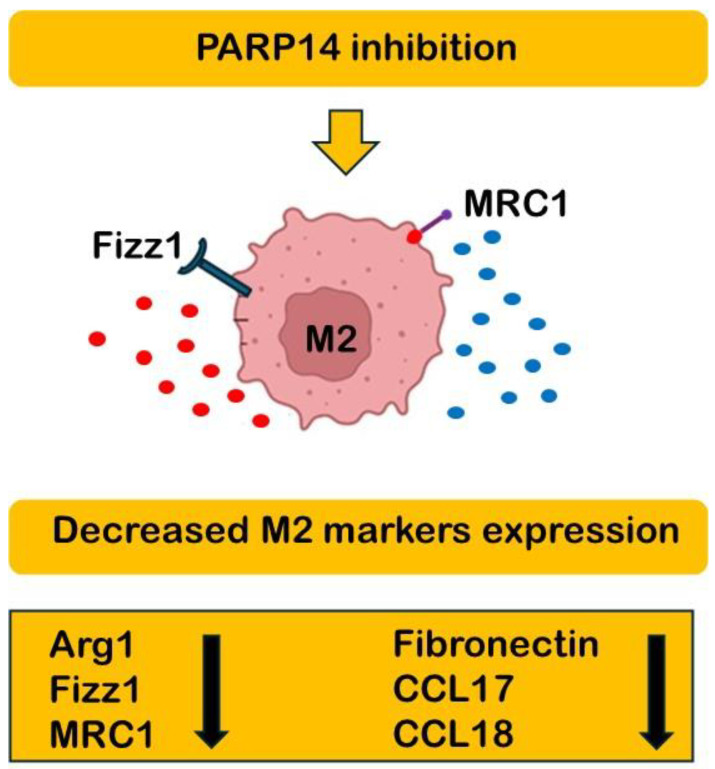
Schematic representation of the rationale behind PARP14 inhibition to achieve a switch from anti-inflammatory to pro-inflammatory macrophages. Down arrows indicate decreased expression.

**Figure 2:**
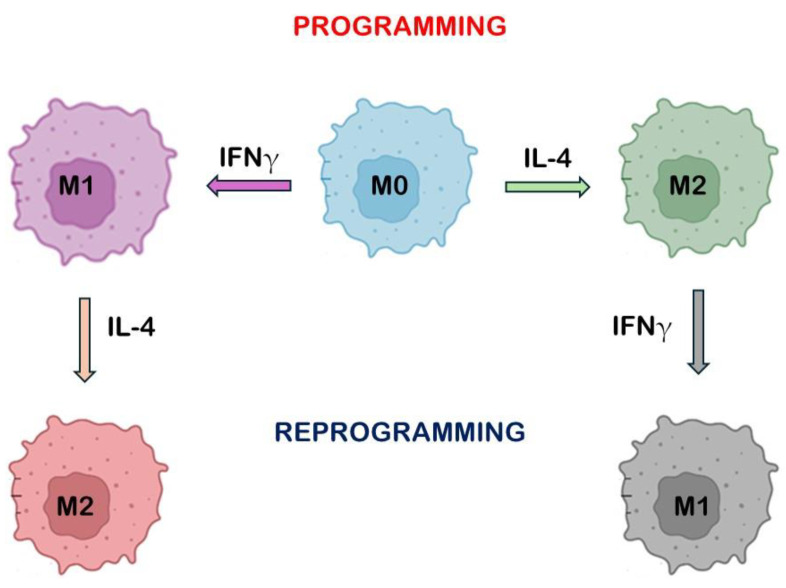
Schematic representation of programming and reprogramming macrophage phenotypes strategy.

**Figure 3:**
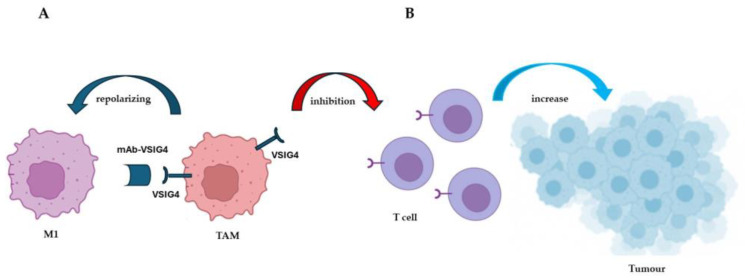
VSIG4 expression is restricted to tumor-associated myeloid populations. (**A**) Monoclonal antibody VSIG4 (mAb-VSIG4) leads to the repolarization of TAMs to an inflammatory M1-like phenotype. (**B**) VSIG4 acts as an inhibitory molecule that suppresses the cells that express it and inhibits T-cell proliferation and cytokine production, promoting tumor growth.

**Figure 4:**
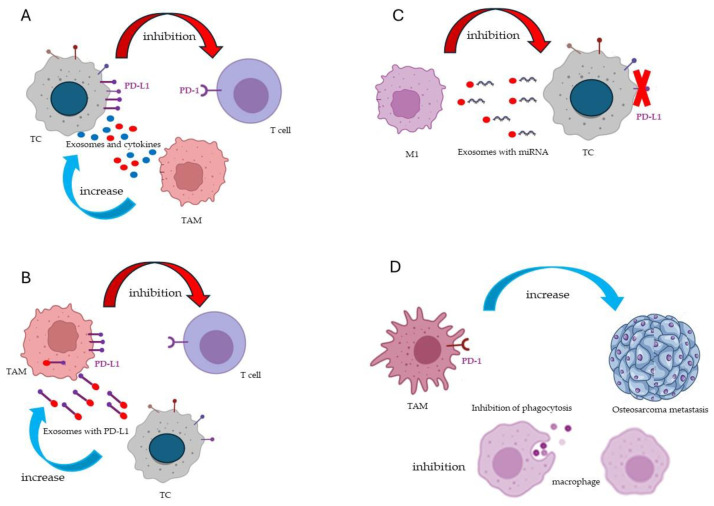
Expression and function of immune checkpoints PD-L1 and PD-1 in macrophages and tumor cells (TCs). (**A**) TAMs can indirectly inhibit immune activation by enhancing PD-L1 expression on TCs through the release of exosomes and anti-inflammatory cytokines. PD-L1 expressed on TCs binds to PD-1 expressed on T cells and inhibits the cytotoxic effects of T cells. (**B**) TAMs can directly inhibit immune activation because they increase their expression of PD-L1 due to the phagocytosis of PD-L1-carrying exosomes produced by TCs. (**C**) M1 macrophages can directly inhibit tumor growth by inhibiting PD-L1 expression in TCs through the release of exosomes carrying miR16-5p. (**D**) PD-1-expressing TAMs inhibit macrophage phagocytosis and promote tumor growth and metastasis. Adopted from [17].
